# Exposure to volatile organic compounds is a risk factor for diabetes retinopathy: a cross-sectional study

**DOI:** 10.3389/fpubh.2024.1347671

**Published:** 2024-01-30

**Authors:** Zhi Wang, Dongjun Chen, Lingling Peng, Xian Wang, Qun Ding, Liang Li, Tongdao Xu

**Affiliations:** ^1^Department of Endocrinology, The Second People’s Hospital of Lianyungang, Lianyungang, China; ^2^Department of Cardiac Function Examine, The Second People’s Hospital of Lianyungang, Lianyungang, China; ^3^Department of Ultrasonography, The Second People’s Hospital of Lianyungang, Lianyungang, China

**Keywords:** diabetes mellitus, diabetic retinopathy, volatile organic compounds, epidemiology, NHANES

## Abstract

**Introduction:**

A few past experimental studies have indicated that exposure to volatile organic compounds (VOCs) might be a potential risk factor for diabetes retinopathy (DR). However, these findings lack substantial support from extensive epidemiological research. This large-scale cross-sectional study aimed to examine whether exposure to low levels of VOCs in the general population is associated with diabetes mellitus (DM) and DR.

**Methods:**

The analytical data was from the National Health and Nutrition Examination Survey (NHANES) dataset (2011–2018). To minimize the potential impact of gender and age on the findings, propensity score matching was utilized to align the data selection. Relationships between blood VOCs and DM and DR were assessed in a sample of 2,932 adults using the logistic regression models. Additionally, Bayesian kernel machine regression (BKMR) models and Weighted Quantile Sum (WQS) were conducted for mixture exposure analysis.

**Results:**

The result shows VOCs were positive associated with DM and DR in US adults, as assessed by WQS model, and the calculated odd ratios (ORs) [95% confidence interval (C.I)] were 53.91(34.11 ~ 85.22) and 7.38(3.65 ~ 14.92), respectively. Among the components of VOCs, 1,2-Dibromoethane, Carbon Tetrachloride and 2,5-Dimethylfuran were positive related with the DR, and ORs (95%C.I) were 2.91(2.29 ~ 3.70), 2.86(2.25 ~ 3.65) and 2.19(1.79 ~ 2.94), respectively. BKMR model shows that there was a dose–response relationship between combined VOCs and DR, although the relationship was non-linearly.

**Conclusion:**

This study suggested that exposure to VOCs may increase the risk of DR, which had important public health implications.

## 1 Introduction

Diabetes mellitus (DM) is a chronic metabolic disorder characterized by high levels of blood glucose due to insufficient insulin production or impaired insulin action (1). According to the World Health Organization (WHO) and the International Diabetes Federation (IDF), in 2021, approximately 537 million adults aged 20 to 79 worldwide are affected by DM, directly contributing to 1.5 million deaths annually (2). The most prevalent complications associated with DM include diabetic nephropathy (DN) (3), diabetic retinopathy (DR) (4), and cardiovascular diseases. These complications have a significant impact on the quality of life and increase the risk of mortality in individuals with diabetes. DR, which is a common complication of DM, can cause visual impairment and blindness on a global scale (5). As the incidence of DM rapidly grows, the prevalence of DR also increases accordingly. Previous studies have shown that the occurrence rate of DR in DM patients worldwide is 34.6% (6). In China, the prevalence of DR is 1.7% in the general population and 22.4% among diabetics (7). The growing population of individuals affected by DR not only demands a substantial allocation of medical resources but also imposes a significant burden on society. Consequently, the prevention and control of DR assume paramount importance in the current era of heightened diabetes prevalence.

In recent years, there has been a surge in awareness regarding the correlation between environmental pollution and human health (8). Extensive scientific research has firmly established that DR is intricately connected to environmental changes. Notably, a study conducted by Fan Cao et al. (9) revealed that exposure to air pollution, such as PM_2.5_/PM_0.1_ and environmental tobacco smoke, significantly increased the occurrence of autoimmune eye diseases, including DR and Graves’ ophthalmopathy. Furthermore, a comprehensive scoping review involving 27 studies found compelling evidence suggesting that air pollution could be a modifiable risk factor for chronic eye diseases (10).

Volatile organic compounds (VOCs) encompass a wide array of chemicals extensively employed as solvents, degreasers, and cleaning agents in both industrial and consumer applications (11). It has been revealed through numerous investigations that a significant number of these VOCs have contaminated groundwater, drinking water sources and indoor areas such as homes, offices and classroom where person spend most her/his time. Previous studies have highlighted the adverse effects of exposure to VOCs on human health (11, 12). A perspective review summarized the impacts of VOCs and considered that prolonged exposure to VOCs might have an effect on various physiological functions of the body, including prostate function, respiratory system, lung function levels, and sex hormone levels (11). Some studies have found that VOCs are a risk factor for DM in the US population (13, 14). However, few study was to investigate the association between VOCs and DR, and there is a lack of epidemiological evidence to support the correlation between DR and VOCs.

The National Health and Nutrition Examination Survey (NHANES) is a cross-sectional study conducted by the Centers for Disease Control and Prevention of the United States. This study collects data on the status of DR, concentrations of VOCs, and the presence of DM in the participants. By analyzing this data, we can investigate the relationship between VOCs and DR.

## 2 Methods

### 2.1 Study design and participants

**Figure 1:**
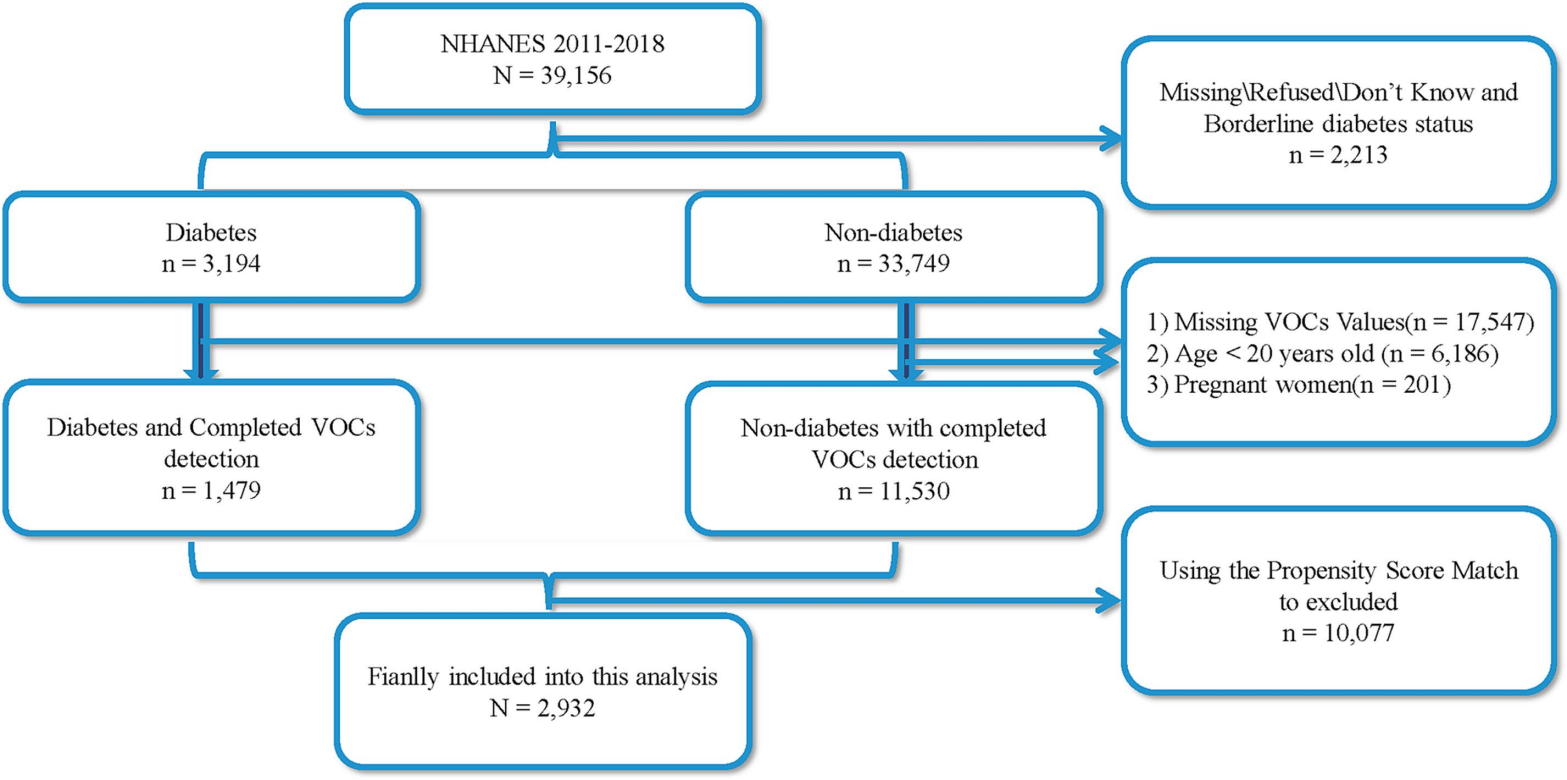
The flowchart of finally analysis participants.

The data of this cross-sectional was from the National Health and Nutrition Examination Survey (NHANES, https://www.cdc.gov/nchs/nhanes) 2011–2018. A total of 39,156 participants were recruited from four rounds of NHANES, and all participants was divided into two groups based on whether participants were diagnosed with diabetes: 3,194 cases of diabetes patients and 33,749 cases of non-diabetes patients, with 2,213 missing cases. Among all individuals, a total of 6,186 participants under the age < 20 years old, 201 women with pregnant, and 17,547 participants who had not completed the volatile organic compound metabolites (mVOCs) detection were excluded. Ultimately, 1,479 cases of diabetes patients and 11,530 cases of non-diabetes patients were included. Considering that age and gender are influencing factors for diabetes, this study utilized the PSM score method to match 1,479 cases of diabetes patients with 11,530 non-diabetes participants. A successful match was made for 1,466 pairs of patients. Therefore, a total of 2,932 patients were included in this study, with 1,466 cases of diabetes patients and 1,466 cases of non-diabetes patients (Figure 1). Among the diabetes patients, 321 individuals with diabetes and DR. All participants with this cross-section study were more than 20 years old, and the informed consents and ethical approval could be found on the web of NHANES.

### 2.2 Measurement of whole blood mVOCs

The measurement of mVOCs in human whole blood was carried out by capillary gas chromatography and mass spectrometry with selected-ion monitoring detection and isotope-dilution. This method quantifies levels of individual mVOCs and Trihalomethanes and methyl tert-butyl ether in whole blood to low-parts-per-trillion range. Because non-occupationally exposed individuals have blood mVOC concentrations within this range, this method is applicable for determining these quantities and investigating cases of sustained or recent low-level exposure. Detailed methods and information of mVOCs could be found on the web of NHANES. To ensure data integrity and sufficient sample size, we carefully selected 22 mVOCs (In the NHANES surveys conducted between 2011 and 2018, a total of 32 mVOCs were detected in the 2011–2012 cycle, 39 VOCs in the 2013–2014 cycle, 40 VOCs in the 2015–2016 cycle, and 40 VOCs in the 2017–2018 cycle. To increase the sample size, we combined the data from all four cycles and focused on VOCs that were consistently detected across all four cycles. As a result, a total of 22 VOCs were identified in the intersection of the four cycles, and these 22 VOCs were chosen for further analysis). The 22 mVOCs included 2,5-Dimethylfuran, 1,1,1,2-Tetrachloroethane, Hexane, 1,2-Dichlorobenzene, 1,2-Dichloroethane, 1,3-Dichlorobenzene, Tetrachloroethene, Benzene, Chlorobenzene, Carbon Tetrachloride, 1,4-Dichlorobenzene, 1,2-Dibromoethane, Ethylbenzene, Furan, Isopropylbenzene, Methylene Chloride, Nitrobenzene, o-Xylene, Trichloroethene, 1,1,1-Trichloroethane, 1,2,3-Trichloropropane and m−/p-Xylene.

### 2.3 Measurements of DM and DR

In the NHANES survey, there is a questionnaire specifically designed to inquire whether the participants have diabetes. The question is as follows: “{Have you/Has SP} ever been told by a doctor or health professional that {you have/{he/she/SP} has} diabetes or sugar diabetes?” If the participants respond affirmatively with a “yes,” they are considered individuals with diabetes. Another question pertaining to diabetes-related complications is as follows: “Has a doctor ever told {you/SP} that diabetes has affected {your/his/her} eyes or that {you/s/he} had retinopathy?.” This question aims to identify if diabetic individuals have experienced any eye-related issues. If the participants respond positively with a “yes,” they are considered individuals with diabetic retinopathy.

### 2.4 Covariable

In this cross-sectional study, covariates, that were included in the finally analysis, were based on previous studies associated with diabetes. Age, sex, race/ethnicity and education levels were from the demographic data, body mass index (BMI) was collected from examination data.

### 2.5 Statistical analysis

In this study, we utilized propensity score matching (PSM) to select our study subjects. We included a total of 1,479 diabetic patients from NHANES 2011–2018. Considering the impact of age and gender on both VOCs concentrations and diabetes, we carefully matched non-diabetic patients with the diabetes group in terms of age and gender at a comparable ratio. Descriptive analysis of the data was conducted to describe the mean, proportions, and median of demographic information, mVOCs concentrations, BMI, FPG, and fasting insulin levels across different groups. The difference between the three groups was analyzed using the Chi-square test, Mann–Whitney test, and one-way ANOVA. Univariate logistic regression and weighted quantile sum (WQS) were employed to explore the association between DM and DR and individual or combined mVOCs. To investigate the joint effects of mixed pollutants between mVOCs and DM and DR, the study introduced the Bayesian kernel machine regression (BKMR) model to explore the dose–response functions. All data analyses were performed using R software (Version 4.1.3), and *p*-values <0.05 were considered statistically significant.

## 3 Results

### 3.1 The basic characteristic of participants

In Table 1, the characteristics of the participants and the levels of mVOCs were examined in NHANES 2011–2018. The total number of participants was 2,932, consisting of 1,466 non-diabetic individuals, 1,145 individuals with diabetes but without diabetic Retinopathy (DR), and 321 individuals with diabetes and DR. Individuals with diabetes exhibited higher body mass index (BMI) and fasting insulin levels compared to non-diabetic individuals. However, there were no significant differences in these aspects between individuals with diabetes and those with DR. Additionally, race and education levels showed significantly variations among the three groups. Regarding the mVOCs, certain components including 2,5-Dimethyfuran, 1,1,1,2-Tetrachloroethane, Benzene, Carbon Tetrachloride, 1,2-Dibromoethane, Isopropylbenzene, Nitrobenzene, and 1,2,3-Trichloropropane exhibited significant differences among the three groups. While, the levels of 14 other components did not show any noticeable distinctions.

**Table 1:** The clinical characteristic of participants.

Variables	Non-diabetes	Diabetes with non-diabetic retinopathy	Diabetes with diabetic retinopathy	*p*
*n*	1,466	1,145	321	
Sex (male)	770	583	187	0.067
Age (years)	61.80 ± 12.61	61.50 ± 12.81	62.85 ± 11.80	0.237
Diabetic duration (years)	~~	8.00 (4.00 ~ 15.00)	15.00 (7.50 ~ 24.50)	<0.001
BMI (kg/m2)	28.08 ± 6.17	32.34 ± 7.43	31.67 ± 6.88	<0.001
FPG (mmol/L)	5.78 ± 1.19	8.72 ± 3.42	9.03 ± 4.23	<0.001
Fasting insulin (uU/mL)	9.63 (6.15 ~ 14.79)	12.92 (7.94 ~ 22.33)	10.73 (7.74 ~ 23.24)	<0.001
*Race*				
Mexican American	127 (8.7%)	196 (17.1%)	52 (16.2%)	<0.001
Other Hispanic	129 (8.8%)	104 (9.1%)	37 (11.5%)	
Non-Hispanic White	614 (41.9%)	370 (32.3%)	100 (31.2%)	
Non-Hispanic Black	378 (25.8%)	306 (26.7%)	83 (25.9%)	
Non-Hispanic Asian	193 (13.2%)	133 (11.6%)	38 (11.8%)	
Other race	25 (1.7%)	36 (3.1%)	11 (3.4%)	
*Education level*				
Less than 9th grade	177 (12.1%)	184 (16.1%)	54 (16.8%)	0.001
9-11th grade	195 (13.3%)	160 (14.0%)	51 (15.9%)	
High school graduate	329 (22.4%)	267 (23.3%)	82 (25.5%)	
Some college or AA degree	384 (26.2%)	303 (26.5%)	78 (24.3%)	
College graduate or above	381 (26.0%)	231 (20.2%)	56 (17.4%)	
*Volatile organic compounds*				
2,5-Dimethylfuran (ng/L)	7.80 (7.80 ~ 26.80)	8.00 (7.80 ~ 8.00)	8.00 (7.80 ~ 8.00)	0.036
1,1,1,2-Tetrachloroethane (ng/L)	28.30 (28.30 ~ 28.30)	28.00 (28.00 ~ 28.30)	28.00 (28.00 ~ 28.30)	<0.001
Hexane (ng/L)	86.30 (86.30 ~ 86.30)	86.20 (86.00 ~ 86.30)	86.20 (86.00 ~ 86.30)	0.638
1,2-Dichlorobenzene (ng/L)	17.70 (17.70 ~ 17.70)	18.00 (17.70 ~ 18.80)	18.00 (17.70 ~ 18.00)	0.926
1,2-Dichloroethane (ng/L)	7.10 (7.10 ~ 7.10)	7.08 (7.00 ~ 7.10)	7.08 (7.00 ~ 7.10)	0.631
1,3-Dichlorobenzene (ng/L)	17.70 (17.70 ~ 17.70)	18.00 (18.00 ~ 18.00)	18.00 (17.70 ~ 18.00)	0.074
Tetrachloroethene (ng/L)	33.90 (33.90 ~ 55.00)	34.00 (33.90 ~ 34.00)	34.00 (33.90 ~ 34.00)	0.392
Benzene (ng/L)	17.00 (17.00 ~ 57.39)	17.00 (17.00 ~ 52.00)	17.00 (17.00 ~ 44.00)	0.023
Chlorobenzene (ng/L)	7.80 (7.80 ~ 7.80)	8.00 (7.80 ~ 8.00)	8.00 (7.80 ~ 8.00)	0.126
Carbon Tetrachloride (ng/L)	3.50 (3.50 ~ 3.50)	4.00 (3.50 ~ 4.00)	4.00 (3.50 ~ 4.00)	<0.001
1,4-Dichlorobenzene (ng/L)	65.50 (28.30 ~ 633.50)	42.00 (28.00 ~ 273.00)	63.00 (28.30 ~ 542.00)	0.366
1,2-Dibromoethane (ng/L)	10.60 (10.60 ~ 10.60)	11.00 (10.60 ~ 11.00)	11.00 (10.60 ~ 11.00)	<0.001
Ethylbenzene (ng/L)	17.00 (17.00 ~ 48.65)	17.00 (17.00 ~ 36.00)	17.00 (17.00 ~ 28.00)	0.470
Furan (ng/L)	17.00 (17.00 ~ 27.14)	18.00 (17.70 ~ 18.00)	18.00 (17.70 ~ 18.00)	0.129
Isopropylbenzene (ng/L)	28.30 (28.30 ~ 28.30)	28.00 (28.00 ~ 28.30)	28.00 (28.00 ~ 28.32)	0.007
Methylene Chloride (ng/L)	176.80 (176.80 ~ 176.80)	177.00 (176.80 ~ 177.00)	177.00 (176.80 ~ 177.00)	0.451
Nitrobenzene (ng/L)	226.30 (226.30 ~ 226.30)	226.00 (226.00 ~ 226.30)	226.00 (226.00 ~ 266.30)	<0.001
o-Xylene (ng/L)	17.00 (17.00 ~ 38.58)	17.00 (17.00 ~ 32.00)	17.00 (17.00 ~ 28.00)	0.410
Trichloroethene (ng/L)	8.50 (8.50 ~ 8.50)	8.00 (8.00 ~ 8.50)	8.00 (8.00 ~ 8.50)	0.658
1,1,1-Trichloroethane (ng/L)	7.10 (7.10 ~ 7.10)	7.06 (7.00 ~ 7.10)	7.00 (7.00 ~ 7.10)	0.553
1,2,3-Trichloropropane (ng/L)	28.30 (28.30 ~ 28.30)	28.00 (28.00 ~ 28.30)	28.00 (28.00 ~ 28.30)	<0.001
m−/p-Xylene (ng/L)	72.00 (40.50 ~ 123.78)	49.00 (24.00 ~ 110.00)	50.00 (24.00 ~ 96.00)p	0.219

BMI, body mass index; FPG, fasting plasma glucose.

### 3.2 Association of the risk of DM and DR with single VOCs levels and union mVOCs

**Figure 2:**
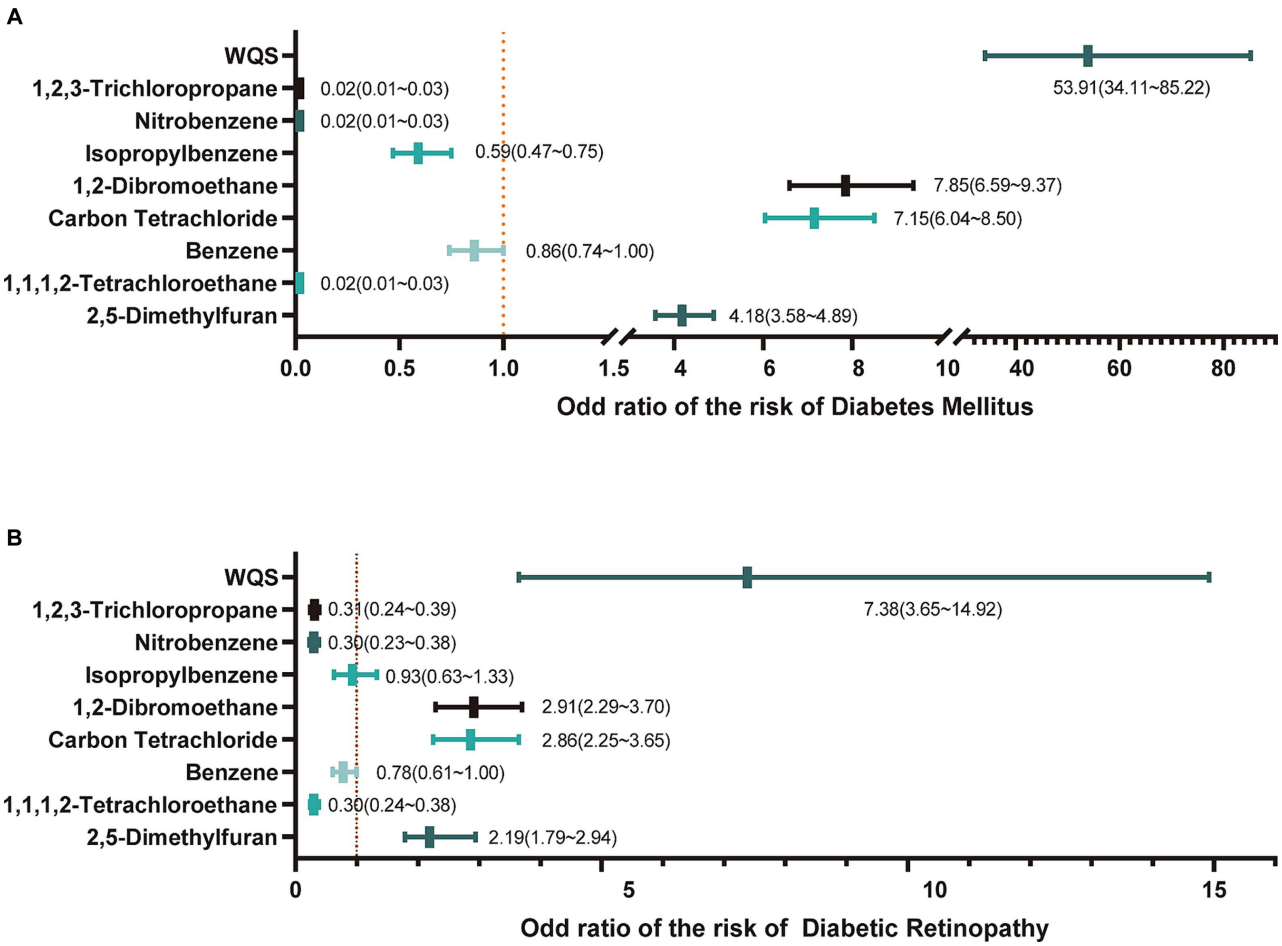
The relationship of mVOCs with diabetes mellitus and diabetic retinopathy. **(A)** The odd ratio of mVOCs on the risk of diabetes mellitus. WQS, weighted quantile sum; **(B)** The odd ratio of mVOCs on the risk of diabetic retinopathy.

In the initial step, we observed distinct levels of eight VOC components among the three groups. Subsequently, logistic regression was employed to examine the relationship between individual VOCs and the risk of DM and DR. Additionally, the WQS was utilized to assess the association between combined VOC levels and the risk of DM and DR development. Prior to analysis, the data type of VOCs was transformed into a binary variable based on the respective median values. In Figure 2A, we observed a negative correlation between the risk of DM and the presence of 1,2,3-Trichloropropane, Nitrobenzene, Isopropylbenzene, and 1,1,1,2-Tetrachloroethane, and the odds ratios (ORs) and 95% confidence interval (C.I) were 0.02(0.01 ~ 0.03), 0.02(0.01 ~ 0.03), 0.59(0.47 ~ 0.75) and 0.02(0.01 ~ 0.03), respectively. Conversely, we found a positive association between the risk of DM and the presence of 1,2-Dibromoethane, Carbon Tetrachloride, and 2,5-Dimethylfuran, and ORs and 95%C.I were 7.85(6.59 ~ 9.37), 7.15(6.04 ~ 8.50) and 4.18(3.58 ~ 4.89), respectively. Furthermore, the WQS indicated that higher levels of combined mVOCs were positively associated with an increased risk of DM after adjusting for sex, age, education levels, race/ethnicity and BMI, with an OR of 53.91 (95% C.I,34.11 ~ 85.22). In Figure 2B, we found that there was a negative association of the risk of DR with the presence of 1,2,3-Trichloropropane, Nitrobenzene and 1,1,1,2-Tetrachloroethane, and ORs (95%C.I) were 0.31(0.24 ~ 0.39), 0.30(0.23 ~ 0.38) and 0.30(0.24 ~ 0.38), respectively. 1,2-Dibromoethane, Carbon Tetrachloride and 2,5-Dimethylfuran were positive related with the risk of DR, and ORs (95%C.I) were 2.91(2.29 ~ 3.70), 2.86(2.25 ~ 3.65) and 2.19(1.79 ~ 2.94), respectively. There was no significant relationship of Benzene and Isopropylbenzene with the risk of DR. We also explored the association between the combined mVOCs and the risk of DR using WQS after adjusting for sex, age, education levels, race/ethnicity and BMI, and found that there was a positive association with OR of 7.38 (95%CI,3.65 ~ 14.92).

### 3.3 The exposure-response effect of VOCs on DM and DR

**Figure 3:**
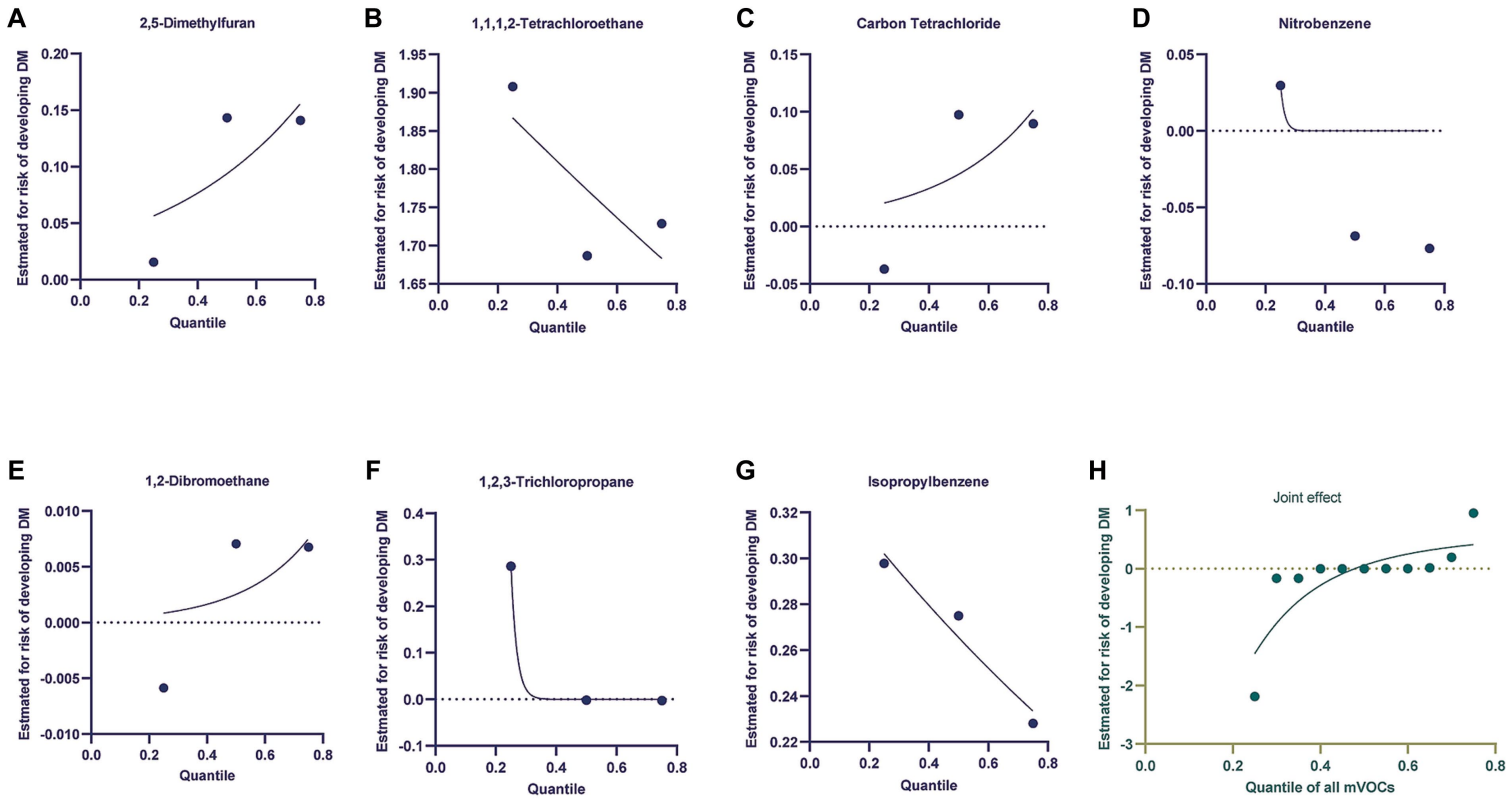
Using the Bayesian kernel machine regression (BKMR) model to investigate the exposure-response functions between mVOCs and diabetes mellitus. **(A–G)** Depicted the individual VOCs and their corresponding exposure-response functions in relation to diabetes mellitus. **(H)** The joint effect of mVOCs on diabetic mellitus.

**Figure 4:**
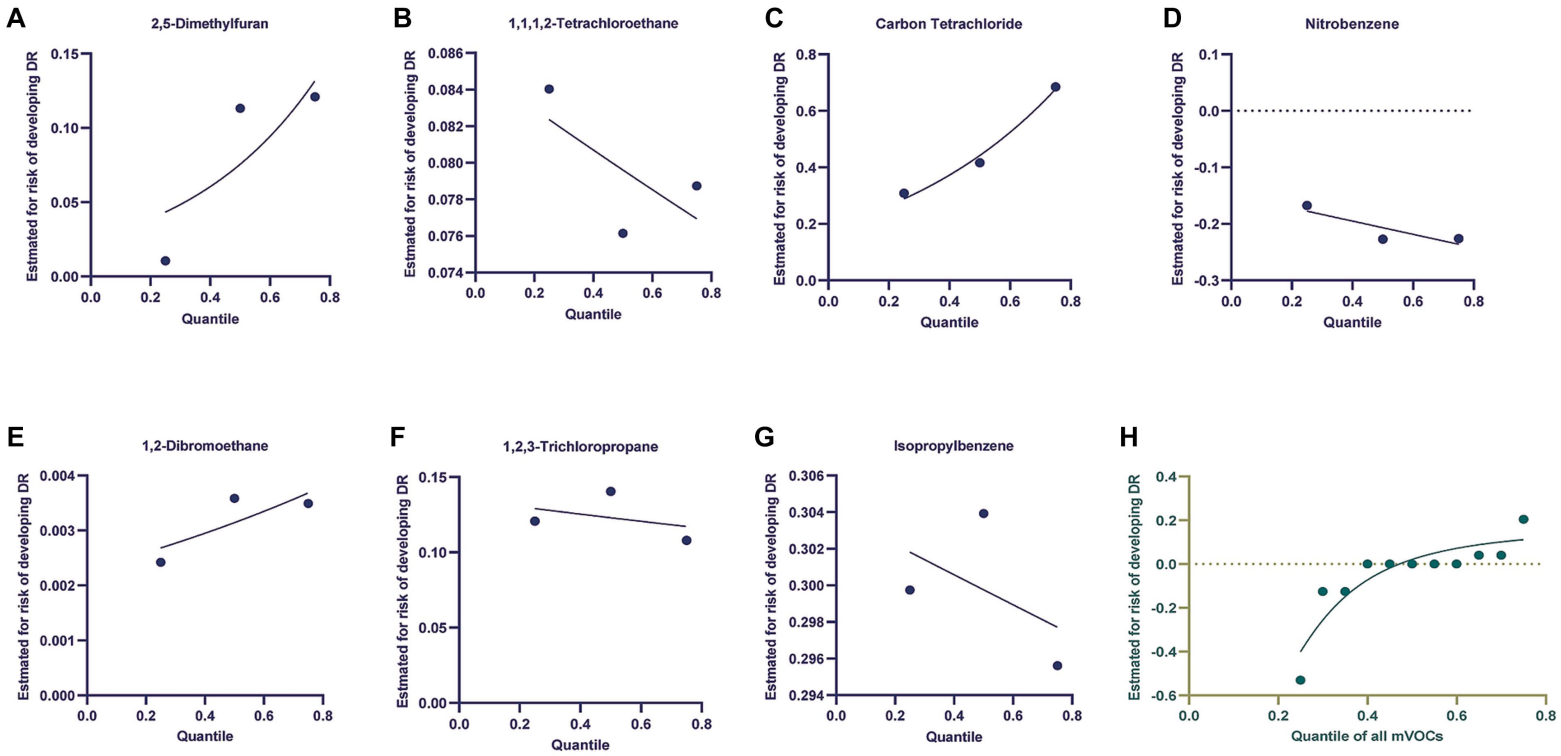
Using the Bayesian kernel machine regression (BKMR) model to investigate the exposure-response functions between mVOCs and diabetic retinopathy. **(A–G)** Depicted the individual VOCs and their corresponding exposure-response functions in relation to diabetic retinopathy. **(H)** The joint effect of mVOCs on diabetic retinopathy.

The BKMR model was utilized to examine the relationship between mVOCs and the development of DM or DR. The model was adjusting for sex, age, BMI, education levels and race/ ethnicity. When observing Figure 3, it was discovered that the likelihood of DM escalated as the level of mVOCs increased. Furthermore, the individual exposures to 2,5-Dimethylfuran, Carbon Tetrachloride, and 1,2-Dibromoethane exhibited a positive correlation with the risk of DM. Negative exposure-response association was observed between 1,1,1,2-Tetrachloroethane, Nitrobenzene, 1,2,3-Trichloropropane, Isopropylbenzene and the risk of DM. Figure 4 showed the univariate and joint exposure-response functions of mVOC and DR. A significant positive exposure-response relationship was observed between 2,5-Dimethylfuran, Carbon Tetrachloride, 1,2-Dibromoethane and the risk of DR. Negative exposure-response association was observed between 1,1,1,2-Tetrachloroethane, Nitrobenzene, 1,2,3-Trichloropropane, Isopropylbenzene and the risk of DR.

## 4 Discussion

In this comprehensive cross-sectional epidemiological study, we have identified an exposure-response relationship between VOCs and both DM and DR. The findings of this study provide epidemiologic evidence supporting a significant association between VOCs and the increased risk of DR. Although VOCs are widely recognized as common environmental pollutants with known adverse effects on the body, their connection to diabetes and its related DR has remained unclear, necessitating further investigation. In this present study, the WQS regression and BKMR model were suggested that the joint effect of VOCs was positive associated with DM and DR.

Several prior studies have attempted to elucidate the particular mechanisms through which environmental pollutants impact DM and its complications (15), insulin resistance (16) and plasma fasting glucose levels (17). However, numerous unexplored mechanisms still remain in need of investigation. Specifically, how environmental pollutants contribute to retinal microvascular changes and neuropathy in individuals with diabetes. A review conducted by Rao et al. summarized the relationship between air pollution and type 2 diabetes. It highlighted the experimental studies supporting this association, specifically focusing on tissues involved in the pathogenesis of T2DM, such as the immune system, adipose tissue, liver, and central nervous system (18). The development of DR is an intricate process involving a multitude of molecules and biochemical mechanisms (19). Research has indicated that the activation of protein kinase C (20), small GTP-binding proteins (21), and the mitogen-activated protein kinase pathway (22), along with the responses of oxidative stress (23) and endoplasmic reticulum stress, are all mechanisms associated with and contributing to the onset of DR. In these mechanisms, oxidative stress should be given sufficient attention. Research shows that the retina is rich in polyunsaturated fatty acids, has a high capacity for oxygen intake and glucose consumption, and is more susceptible to damage from oxidative stress compared to other tissues (24). Chen et al. (25) conducted a study involving 454 patients diagnosed with gestational diabetes mellitus (GDM) and 454 healthy controls. The researchers measured the concentrations of urinary VOCs as well as oxidative stress markers (8-OHdG, 8-OHG, and HNEMA) for each participant and the findings unveiled a noteworthy positive association between VOCs and oxidative stress markers, implying the involvement of VOCs in the body’s response to oxidative stress.

In fact, numerous components of VOCs exhibit a strong correlation with oxidative stress (26). In several experimental settings, Carbon Tetrachloride (CCl4) is commonly employed as an agent to induce liver injury (27). This is attributed to CCl4 undergoing metabolism in the liver through cytochrome P450 superfamily monooxygenases (CYP family), resulting in the generation of trichloromethyl free radical (CCl3*). Subsequently, those free radical initiates lipid peroxidation reactions, leading to the production of peroxides that specifically target mitochondria and inflict damage upon hepatocyte membranes. Ultimately, this damaging process contributes to the development of liver fibrosis (28). In our current study, we have discovered a positive correlation between CCl4 and the risk of DR. This finding indicates that CCl4 induces oxidative stress in the retina and elevates the likelihood of DR. The European Food Safety Authority (29) conducted an assessment on the safety of furan and methylfurans in food. Their findings revealed that 2,5-Dimethylfuran exhibits hepatotoxicity in rats, with various mechanisms being implicated including oxidative stress, gene expression modifications, epigenetic fluctuations, inflammation, and enhanced cell proliferation. Pal et al. (30). conducted a comprehensive study to assess the fluctuations in the concentrations of 38 VOCs in urine samples collected from a group of 19 healthy individuals over a 44-day period. Their findings revealed significant and positive correlations between the concentrations of VOCs and oxidative stress biomarkers, including lipid, protein, and DNA damage. However, in our findings, some compounds of VOCs have a negative relationship with the risk of DR, such as 1,1,1,2-Tetrachloroethane, Nitrobenzene, 1,2,3-Trichloropropane and Isopropylbenzene. This phenomenon suggests that the association between VOCs and DR is not a straightforward one. As the concentration of a particular pollutant rises within the body, the body initiates compensatory responses to counteract and mitigate the adverse impacts brought about by that pollutant. Moreover, it is inadequate to assess the individual effects of VOCs components on the human body separately, given that people in the real world are exposed to a myriad of pollutants. Instead, it is essential to evaluate the cumulative impact of a specific category of pollutants on human health. In this study, we observed a positive correlation between the concentration of VOCs extracted from blood samples and the risk of DR, and our study employed propensity score matching to mitigate the influence of age and gender on the findings, and the analysis of the overall impact of VOCs using WQS and BKRM models is also deemed reliable. In our results, we found that race and education levels were significantly difference among three groups. It is well known that race was a risk factor of DM and DR. DM is a multifaceted condition that is influenced by a combination of environmental and genetic factors. The diverse genetic backgrounds present in different ethnic groups can contribute to varying incidence rates of DM (31). In general, there is a positive correlation between educational background, economic income, and cultural level (32). Individuals with higher levels of education are more likely to be aware of the potential risks that diseases can pose and have greater resources to prevent and control them.

Some previous studies reported the association between VOCs and the risk of DM using NHANES dataset (13, 14), however, few study was to explore the relationship between VOCs and the risk of DR. We hypothesize that there could be multiple factors contributing to this observation. Firstly, the number of individuals with DR in the NHANES database is relatively limited compared to the overall diabetic population, which is a constraint of this study. Secondly, DR is recognized as a complication of diabetes, and if there is evidence linking VOCs to diabetes, it is plausible that VOCs may indirectly influence the occurrence and progression of DR through their impact on blood glucose levels. Nevertheless, we acknowledge that susceptibility to DR may also play a role, necessitating further comprehensive investigation.

Some limitations should be stated in this study. Firstly, it should be noted that this is a cross-sectional analysis, which means we cannot verify the causal association between exposure to VOCs and the risk of DR. Secondly, it is to mention that the participants in this study were solely Americans, and thus our findings need further validation in other countries. Finally, we used the propensity score matching to reduce the confounding effect of sex and age, but this method may result in sample attrition. Nonetheless, we believe that using propensity score matching in cross-sectional studies is still necessary.

## 5 Conclusion

We conducted a comprehensive investigation into the relationship between multiple blood VOCs and DM and DR using data from the NHANES 2011–2018. A total of 2,932 US adults with complete data participated in this study. Our analyses, utilizing WQS and BKMR models, revealed a positive association between multiple blood VOCs and both diabetes and DR. Notably, increased concentrations of CCl4 were significantly and positively correlated with the presence of DR. Furthermore, co-exposure to multiple VOCs exhibited a positive correlation with DR, with the majority of VOCs demonstrating a nonlinear relationship with diabetes and DR. While our study suggests a connection between VOCs exposure and DR, more evidence is required to substantiate these findings.

## Data availability statement

The original contributions presented in the study are included in the article/supplementary material, further inquiries can be directed to the corresponding authors.

## Ethics statement

The studies involving humans were approved by the ethics committee of the second people’s hospital of Lianyungang. The studies were conducted in accordance with the local legislation and institutional requirements. The participants provided their written informed consent to participate in this study.

## Author contributions

ZW: Investigation, Methodology, Writing – original draft. DC: Data curation, Software, Validation, Visualization, Writing – review & editing. LP: Formal analysis, Visualization, Writing – review & editing. XW: Investigation, Software, Visualization, Writing – review & editing. QD: Investigation, Software, Visualization, Writing – review & editing. LL: Conceptualization, Methodology, Project administration, Visualization, Writing – original draft, Writing – review & editing. TX: Conceptualization, Project administration, Writing – review & editing.
